# biDCG: A New Method for Discovering Global Features of DNA Microarray Data via an Iterative Re-Clustering Procedure

**DOI:** 10.1371/journal.pone.0102445

**Published:** 2014-07-21

**Authors:** Chia-Pei Chen, Hsieh Fushing, Rob Atwill, Patrice Koehl

**Affiliations:** 1 Department of Statistics, University of California Davis, Davis, California, United States of America; 2 Department of Population, Health and Reproduction/Vet Med Extension, University of California Davis, Davis, California, United States of America; 3 Department of Computer Science and Genome Center, University of California Davis, Davis, California, United States of America; University of California, Riverside, United States of America

## Abstract

Biclustering techniques have become very popular in cancer genetics studies, as they are tools that are expected to connect phenotypes to genotypes, i.e. to identify subgroups of cancer patients based on the fact that they share similar gene expression patterns as well as to identify subgroups of genes that are specific to these subtypes of cancer and therefore could serve as biomarkers. In this paper we propose a new approach for identifying such relationships or biclusters between patients and gene expression profiles. This method, named biDCG, rests on two key concepts. First, it uses a new clustering technique, DCG-tree [Fushing et al, PLos One, 8, e56259 (2013)] that generates ultrametric topological spaces that capture the geometries of both the patient data set and the gene data set. Second, it optimizes the definitions of bicluster membership through an iterative two-way reclustering procedure in which patients and genes are reclustered in turn, based respectively on subsets of genes and patients defined in the previous round. We have validated biDCG on simulated and real data. Based on the simulated data we have shown that biDCG compares favorably to other biclustering techniques applied to cancer genomics data. The results on the real data sets have shown that biDCG is able to retrieve relevant biological information.

## Introduction

Before the “-omics” revolutions, cancer detection and diagnosis relied mostly on changes in phenotypes. Clinicians would identify and categorize cancer cells based on differences in appearance under a microscope compared to equivalent normal cells, namely according to their pathology. The advents of genomics and proteomics at the end of last century however have opened the doors to molecular diagnostics of cancer by providing the tools to study directly all the genes and proteins in a cell [1]–[7]. By studying gene expression patterns in different types of cells (normal, pre-cancerous, and cancerous with difference types and at different stages), molecular diagnostics aim at uncovering “molecular signatures”, i.e. those expression patterns that are specific to a pathology. DNA microarrays, also called “gene chips” or “DNA chips”, make success of this approach possible as they allow researchers to monitor the expression of thousands to hundreds of thousand genes at once [8], [9]. A large number of studies have been published over the last decade that attempt to classify and explain several human diseases on the basis of gene expression data obtained on groups of diseased and healthy subjects. Interestingly, while the technologies behind the DNA chips used in these studies are now quite mature, the methods for processing [10] and analyzing the data they generate have not yet converged to a consensus approach and there are still many new techniques that are proposed. In this paper, we are concerned with the latter.

A set of objects 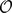, each characterized by some measured features 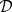, is typically analyzed using clustering, a data analysis technique that performs grouping such that objects in the same group are more similar than objects in the other groups, where similarity is defined by comparisons of the features. As such, clustering techniques are at the core of many data science disciplines, including pattern recognition, knowledge discovery, and classification; their applications to studying data derived from DNA microarray experiments seem therefore quite natural. Microarray data however are somewhat special in that this clustering analysis can usually be performed in two ways [11], [12]. Let us consider for example a microarray experiment designed to differentiate different cancer types. In such an experiment the expression levels of 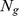 genes are recorded over 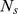 samples, i.e. tissues extracted from a large group of healthy and diseased subjects, leading to an expression matrix of size 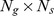. A typical experiment would have 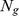 in the order of several thousands and 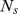 in the order of tens to hundreds. A first approach to analyze these expression patterns is to consider the 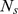 samples as objects and the 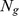 genes as features. Clustering would then regroup samples based on the similarities of their gene expression patterns, hopefully leading to groups that can be identified with cancer types, with one additional group for the healthy subjects. Conversely, the 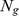 genes can be considered as the objects with the 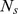 samples becoming the features. Clustering would then identify subsets of genes with similar expression patterns in the different cells under study, with each subset hopefully involving genes that are biologically related to the same mechanistic pathway. Many methods have been developed that allow for the two-way analysis of such data matrices, most of whom specifically for microarray data. These methods are usually referred to as biclustering, or co-clustering. It is beyond the scope of this paper to provide an complete overview of these methods and we refer the readers to this non exhaustive list of excellent reviews and comparison studies [13]–[27]. In the following, we restrict ourselves to describing techniques relevant to our new method.

The first approaches for analyzing microarray data either clustered genes only based on expression patterns [11], or clustered genes and samples independently, with the expression data matrix being subsequently reorganized according to the corresponding trees [12]. While the latter approach showed promising results in separating cancerous from non cancerous tissues as well as for identifying organization in gene expression in these tissues, it does not take into account correlations between genes and samples. For example, it would not reveal if a gene is involved in more than one biological process. In addition, it clusters the samples based on the expression patterns of all genes, while only a few may be relevant to a specific subgroup; the other genes would then be seen as noise that would affect the quality of the clustering results. Ideally, clustering microarray data amounts to identifying sub matrices of the expression matrices, i.e. subsets of rows which exhibit similar behavior for a subset of columns. These submatrices are usually referred to as “biclusters”. Finding the biclusters in an expression matrix usually depends on a merit function that evaluates the quality of these biclusters. Several methods have been developed to solve this NP-hard problem. These methods can be divided into two somewhat opposite groups: those that directly re-organize the rows and columns of the matrix to increase local coherence between samples and genes [13], [21], thereby revealing biclusters, and those that instead narrow down the samples and genes to directly identify stable biclusters, as implemented in the coupled two way clustering (CTWC) method [28]–[30] and in the interrelated two-way clustering (ITWC) method [31]. The new method described in this paper falls in the latter category.

The main rationale behind CTWC and ITWC is noise reduction. By acknowledging that data in the expression matrix are ultimately organized in biclusters (allowing for some data to be outside), they proceed by iteratively constructing subgroups of genes and samples with better signal to noise ratio. Reducing the gene dimension is expected to improve the accuracy of class discovery among the samples, which in turn is expected to guide better grouping of genes. Our approach differs as it is designed to dynamically validate biclusters by looking for consistency in the two-way clustering of the data. Starting with a class 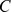 of samples, we cluster the genes by restricting their features to the subgroup 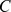. For each cluster of genes 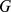, we recluster all samples, limiting their features to the subgroup 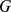. If a resulting cluster of samples contains exclusively members of the input class 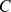, the couple (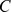, 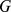) is deemed to be a bicluster; the procedure is then iterated until all stable biclusters are identified.

The key to the success of any of the biclustering methods mentioned above, including our own, is the quality of the clustering algorithm they use. In principle biclustering can be adapted with any one-way clustering method; in practice however, all methods have been optimized with a specific technique, including Hierarchical clustering used by Eisen *et al* [11], a variant of the deterministic annealing algorithm used by Alon *et al* [12], the k-means and fuzzy C-mean algorithms used in variants of the ITWC method [31], [32], and the super magnetic clustering algorithm (SPC) [33], [34] used in CTWC [28]. Our procedure is based on our own new clustering method, referred to as the Data Cloud Geometry (DCG) [35] and its extension that collect the information generated by DCG to generate an ultrametric topological space, which is equivalent to a hierarchical tree, the DCG-tree [36]. This new procedure has two main features that are keys to its success. Firstly, it derives from the empirical similarity measurements a hierarchy of clustering configurations that captures the geometric structure of the data. This hierarchy is then transformed into an ultrametric space, which is less sensitive to noise in the data [36]. Secondly, it has a built-in mechanism for self-correcting clustering membership across different tree levels. These two key features make DCG well suited for two-way analyses of microarray data. We note that DCG-tree is similar in spirit to SPC; its implementation however is simpler and it is more effective computationally. It has been applied to analyze fMRI data [37], as well as to study binary networks [38].

We have applied our biclustering technique based on DCG-tree, which we refer to as biDCG, to simulated as well as real data, the latter derived from experiments on lung cancer [39]. We use these results to illustrate some of the key features of the method, including its robustness with respect to measurement errors and its ability to detect robust biclusters.

This paper is organized as follows. The next section introduces our approach and describes its implementation. The following section presents the results of its applications on simulated and real data. We then conclude with a discussion of future work.

## Methods

### biDCG: Motivation and algorithm

Let us consider a DNA microarray experiment in which the expressions of the same 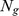 genes have been monitored over a set of 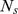 samples. The resulting data are organized in an expression matrix 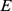 such that 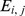 is the intensity (level of expression) associated with gene 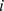 in sample 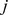. Our goal is to identify partitions of the genes and samples that map with co-regulated families of genes and sub-classes of samples (such as healthy and diseased subjects in the case of cancer-related experiments), respectively. The main difficulties relate to correlations between these partitions, due to the fact that a few genes may be involved in more than one biological process. In addition, a biological process specific to one sample sub-class may only involve a small subset of the genes, in which case the expression levels of the other genes included in the study constitute noise. To circumvent these problems, we align our approach with the concept of coupled biclustering, whose goal is to identify in the expression matrix 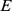 subsets of rows (genes) which exhibit similar behavior for a subset of columns (samples). The complete procedure, which we refer to as biDCG, includes five main steps, namely:

- **step 1**: For a given subclass 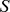 of the samples, construct the DCG-tree on all genes. The features 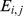 representing the gene 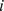 are restricted to the samples 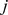 belonging to 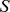.
- **step 2**: Choose a candidate subgroup 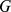 of the genes from a clustering configuration on one level of the computed DCG-tree in Step 1. Construct a DCG-tree for all samples, restricting the features 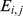 representing a sample 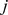 to the genes 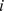 belonging to 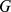.
- **step 3**: Check whether the computed DCG-tree in Step 2 contains a tree branch that only include samples from 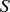, with the rest of the samples being on different and separate branches. If this is true, the pair (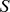,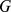) is identified as a bicluster of the expression matrix 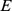. Repeat the Step 2 and 3 for all other subgroups of genes identified in step 1.
- **step 4**: Switch to a different subclass 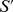, and repeat steps 1 to 3.
- **step 5**: All biclusters (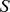,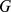) are collected and represented via a specially constructed heat map.

The subclasses of sample considered in step 1 may come from prior knowledge (in which case the clustering is supervised), or from an initial partitioning of the samples using DCG (unsupervised clustering). As described, biDCG is unsupervised and non-parametric in nature. We note that the selection of initial row/column partitioning is not expected to affect the final results, i.e. the definitions of the biclusters: this was observed experimentally on all test cases included in the Results section. The only differences we noticed were the number of iterations needed to reach the stable pattern. Step 3 of this procedure is really a built-in mechanism for assessing the validity of a regrouping of the genes. Step 4 is the actual iterative engine of the algorithm. It is stopped when all subclasses 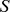 of the samples and all gene subgroups 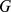 identified in relation to 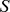 have been analyzed, leading to stable biclusters. The representation obtained in step 5 corresponds to a standard heat map (i.e. a colored matrix 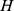 whose element 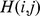 is a square colored upon the intensity 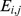, i.e. the level of expression associated with gene 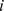 in sample 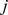) whose rows and columns have been re-organized to identify the bicluster. Namely, to draw a bicluster 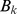 that groups a subset of genes 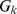 and samples 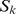, the heat map is reordered so the 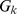 rows and 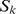 columns appear together. We note finally that the roles of genes and samples can be reversed, i.e. step 1 would start with a subgroup of the genes 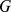, and steps 2 and 3 would cycle through subclasses 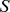 of samples.

### The DCG-tree clustering procedure

A large and complex collection of data, usually called a data cloud, naturally embeds multi-scale characteristics and features, generically termed geometry. Understanding this geometry is the foundation for extracting knowledge from data. We have recently developed a new methodology, called data cloud geometry-tree (DCG-tree) to resolve this challenge [35], [36]. We believe that this DCG-tree procedure is well suited to biclustering as (i), it automatically derives a hierarchy of clustering configurations that captures the geometric structure of the data and therefore does not rely on external parameters, and (ii), it includes a built-in mechanism for self-correcting clustering membership across different tree levels, making it less sensitive to noise. A full description of the DCG-tree method and algorithm is provided in the original papers [35], [36]. We provide a brief outline below as it is essential to understanding the success of biDCG.

Starting from a set of data points and an empirical measure 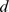 that defines the distances between these data points, our goal is to derive a multi-scale partitioning of these data that illustrates their geometry. The main idea of the DCG method is to embed this geometry into a ferromagnetic potential landscape; its implementation is then based on two key observations. Firstly, it is observed that the empirical distance measure 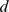 imposes a weighted graph onto the collection of data points (renamed “nodes” in this context). By equating the weight on an edge with a ferromagnetic potential, this weighted graph is seen as equivalent to a potential landscape, typically characterized by many wells with various depths. Secondly, it is possible to explore this landscape and therefore define its geometry by using the popular dynamic Monte Carlo approach. A random walk as a function of “time” will identify the many wells of the potential, as well as the probability of jumping from one well to another. An additional advantage of using dynamic Monte Carlo is that it provides a different dimension to explore the geometry of the landscape, characterized with its temperature parameter 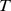. To benefit from the latter, we define the ferromagnetic field such that it places the potential 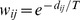 on link 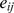 between nodes 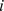 and 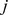 on the graph, where 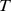 is a parameter mimicking temperature. At a high temperature 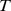, a Markovian walk on the energy landscape will transition from any node to most of the other nodes with more or less equal probabilities. At a low temperature however, the Markov chain tends to get trapped in potential wells for various periods of time depending on the sizes of the well before it can escape. These two observations led to the following two-device algorithm, named Data Cloud Geometry or DCG, for deriving the underlying multi-scale geometry of a data cloud. At a given temperature 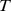, a regulated random walk on the equivalent ferromagnetic landscape as a function of “time” detects information about the number of clusters and the corresponding cluster membership of individual data points. By repeating this procedure at different temperatures, the DCG algorithm derives the geometric hierarchy of the data cloud as follows [35]. First, a range of values for the temperature is defined according to the distribution of experimental distances between the nodes. If computing time is not an issue, then ideally a relatively dense set of initial temperatures is defined within this range. In practice however, a “reasonable” set of temperature is chosen, where reasonable is defined by the computing resources available. For each temperature 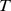, the number 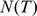 of clusters is then estimated from the corresponding regulated random walk. The plot 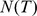 as a function of T reveals a set of critical temperatures [36]. We note that in fact the identification of these critical temperatures is a key feature of DCG and it is an integrand part of its data-driven discovery feature, as these temperatures correspond to major phase-transitions in the data-clustering dynamics. The critical temperatures are then taken as energy barrier heights to define an ultrametric topology onto the data cloud as it is a system at a ground state. This topology provides measurable and natural distances between clusters. The ultrametric topological space can then be summarized as a hierarchical tree, the DCG-tree [36].

There are two main advantages that result from using DCG-tree for biclustering. Firstly, the DCG method is designed to replace the empirical distance measure with an effective ultrametric distance that reflects the underlying structure of the data. This is achieved through the characterization of the field potential built on the links in the data. This ultrametric is much less sensitive to measurement errors. Secondly, the DCG-tree has a built-in mechanism to revise previous clustering decisions.

### Computing distances between vectors of gene expression data

At any step in the biclustering procedure described above, an “object” 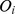 is represented with a vector of expression patterns 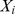 limited to a subset of “features” 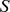 extracted from the expression matrix 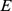, i.e. 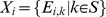. Note that 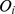 can be a patient, in which case the features are the expression levels of a set of genes for that patient, or 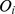 can be a gene, in which case the features are the expression levels of that genes over a set of patients. The simplest measure of similarity between two expression vectors 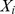 and 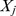 is obtained by computing the Euclidean distance between the two vectors:
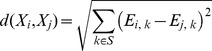



This is the distance measure we will use for the synthetic data in the applications described below. For real data, the Pearson correlation coefficient is usually preferred to the Euclidean distance as it captures the similarity of the expression profiles and ignores differences between the intensities [11], [28]. The use of Pearson's correlation coefficient however relates to a possible linear relationship between two expression profiles; we prefer a less restrictive constraint and use instead the Spearman's correlation coefficient to measure the similarity between two profiles, as the latter only measures the relevance of a monotonic relationship between the two profiles. The Spearman's correlation coefficient 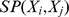 is computed as follows. First, the expression value 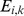 for an object 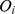 is converted into its rank 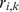 within the vector 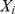. Second, 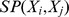 is identified to the Pearson's correlation coefficient of the ranks:
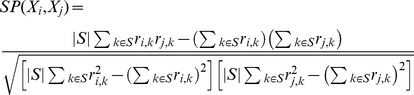



## Results and Discussion

We applied the new iterative re-clustering algorithm biDCG on collections of synthetic and real data sets. The published synthetic data sets have been specifically designed to assess the performances of biclustering techniques [19]; biDCG was used on these sets in an unsupervised way. The real data sets were extracted from published cancer studies [39]. We used these data sets to highlight the ability of biDCG to recover information from data for which satisfactory biological explanation is available. All these experiments (on synthetic and on real data) were performed in an unsupervised way, i.e. without prior knowledge.

### Assessing robustness of biDCG in presence of noise: Synthetic data 1

The first synthetic data we consider were designed by Prelić *et al* [19], following a setting originally proposed by Ihmels *et al* [40], to study the effects of noise in expression matrices with non-overlapping biclusters on the performance of biclustering methods. In this setting, biclusters represent transcription modules; these modules are defined by a set 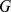 of genes regulated by a set 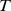 of common transcription factors and a set 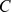 of conditions in which these transcription factors are active. The sizes of 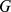, 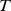, and 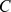 are defined as 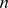, 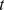, and 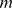, respectively. The transcription modules are defined by two matrices:

- An activation matrix 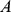 of size 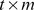 with 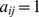 if and only if transcription factor 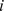 is active in condition 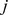;
- A regulation matrix 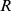 of size 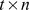 with 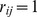 if and only if transcription factor 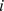 regulates gene 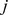.

In the first scenario considered here, 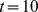 non-overlapping transcription modules, each extending over 10 genes and 5 conditions, emerge. Each gene is regulated by exactly one transcription factor and in each condition only one transcription factor is active. The corresponding data sets are expression matrices 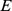 of size 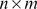 with 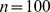 and 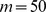 that contain 10 implanted non overlapping biclusters. Two types of expression matrices are considered:

- *Constant biclusters*. The matrix 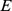 is set according to:
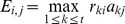
i.e. E is a binary matrix whose elements contained in biclusters are set to 1.
- *Additive biclusters*. The matrix 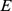 is set according to:
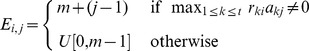
where 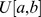 is a uniformly, randomly chosen integer in the interval 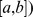. In the resulting matrix, all elements contained in biclusters have a value greater than 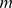, while the remaining elements contain random integer numbers in the range 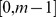.

We note that experiments including the constant biclusters are designed to assess the performance of a biclustering method in identifying subsets of genes with constant expression values within a subset of conditions, according to the terminology introduced by Madeira and Oliveira [16]. In contrast, the additive biclusters are used as a basis to assess the performance of a biclustering method to identify biclusters with coherent values and coherent evolutions.

Noise is simulated by adding random values from a normal distribution to each element of the resulting expression matrices 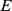. We have considered two levels of noise (i.e. the standard deviation of the normal distribution) for each type of matrices, namely 0.05 and 0.25 for constant biclusters, and 0 and 0.1 for additive biclusters. Note that the latter matrices always contain noise through the function 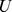, even when the white noise added has a standard deviation of 0.

**Figure 1:**
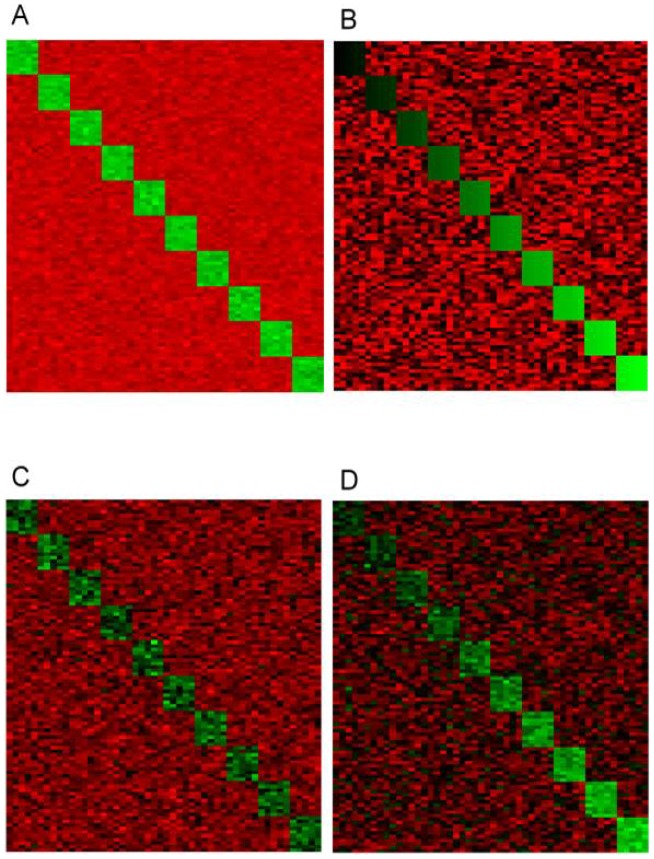
Performance of biDCG on synthetic data representing non-overlapping biclusters. Panels A) and C) show the re-ordered heat maps computed with biDCG based on synthetic expression matrices representing 10 constant non-overlapping biclusters with noise levels 0.05 and 0.25, respectively, while panels (B) and (D) show similar results for additive biclusters with noise levels of 0 and 0.1, respectively. See text for the definition of “constant” and “additive” biclusters.

If there exist a genuine bicluster in a data set, it is expected that this bicluster will be identified as a block in the heatmap after proper permutations on the rows and columns. Figure 1 illustrates that this is indeed observed when applying the biDCG procedure on the two types of expression matrices described above, for two levels of noise. In all cases biDCG recovers correctly the 10 implanted biclusters. We note that the starting conditions (i.e. whether the rows or the columns are considered first) have no impact on the results: for all cases illustrated in Figure 1, the two possible starting conditions led to the same biclusters.

**Figure 2:**
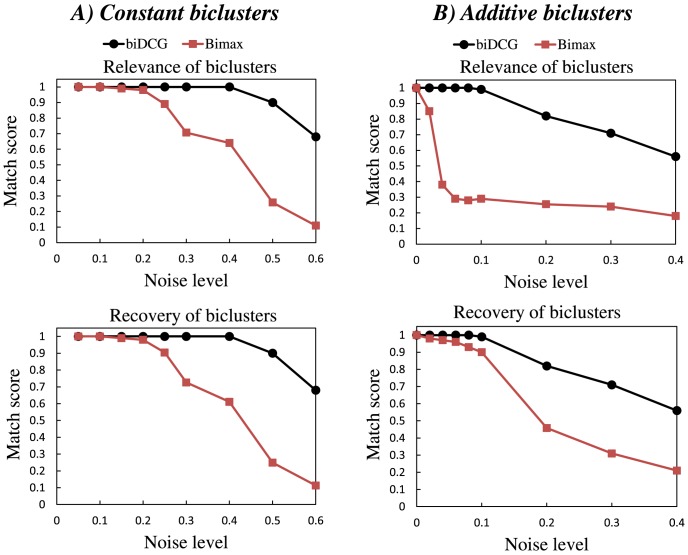
Effects of noise on the relevance and recovery levels of biclusters identified by biDCG and Bimax. The biclustering techniques biDCG and Bimax [19] were applied on synthetic expression matrices designed to represent 10 biclusters, either constant (left panels, A and C), or additive (right panels, B and D). In both cases, the average relevance (i.e. the extent with which a generated bicluster represent a true bicluster) and the average recovery levels (i.e. the extent with which true biclusters are recovered) are plotted as a function of the noise level added to the expression matrices.

To quantify how the performances of biDCG are affected by the presence noise, we use the scores proposed by Prelić and co-workers [19] to measure the performance of our biclustering method. Let 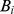 denote the set of implanted biclusters and 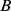 the output of biDCG. The average bicluster relevance 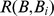 reflects the extent with which the generated biclusters represent true biclusters. In contrast, the average module recovery 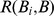 quantifies how well each of the true biclusters is recovered by the biclustering algorithm. A full description of these scores is available in the Supplemental Material of reference [19]. Results for different noise levels for the two types of expression matrices (i.e. with constant or with additive biclusters) are given in Figure 2. For each noise value, 10 different data matrices have been generated from the original gene expression matrix E. The performance of biDCG is averaged over these 10 input matrices. We observe that biDCG is only marginally affected by the presence of noise and recovers more than 98% of all actual biclusters for all noise levels up to 0.1, for both the constant and additive test cases. While all biclusters are correctly recovered at even higher noise levels (up to 0.4) in the constant bicluster case, we observe significantly reduced performance in the additive case for noise levels above 0.1 (for example at a noise level of 0.4, biDCG only recovers 56% of all actual additive biclusters). The poorer performances in the additive case are most likely a consequence of the fact that background noise and biclusters with low expression levels are not clearly separated in the presence of high levels of noise. At even higher noise level (0.4 to 0.6), the performance of biDCG for constant biclusters case is significantly reduced, as reported by both the relevance and recovery scores.

For comparison, we show on Figure 2 the performances of Bimax [19] on the same synthetic data. Bimax, which stands for Binary Inclusion MAXimal biclustering algorithm, uses a fast divide and conquer approach. Expression levels in the gene expression matrix E are first converted to 0 or 1 based to a preset cutoff. The corresponding binary matrix is then divided into two sub matrices U and V by identifying regions that contains a high density of 0 s or 1 s (after row and column rearrangements). The matrices U and V are then sub-divided recursively until no more sub-divisions can be found. The biclusters are then identified with the sub matrices that do no contain only 0 s (see [19] for a full description of the method). While Bimax has known limitations, such as the drawback of possibly missing some good biclusters by early splits, its simplicity and overall successes maintain it as the method of choice for comparison against new biclustering techniques (see for example [27], [41], [42]). Cleary, biDCG outperforms Bimax for both constant and additive clusters at high noise levels. Interestingly, both methods are more robust with respect to noise on the constant clusters than on the additive clusters. The poorer performances on additive clusters are most likely due to the fact that biDCG and Bimax have difficulties when background noise and biclusters are not well separated. The same argument was already mentioned by Prelic and co-workers [19]. Finally we note that since the synthetic data used here are the same as the data used in the comparative studies of Prelic and al., a comparison of Figure 2 in this paper with Figure 2 of reference [19] indicates that biDCG compares well with other biclustering techniques such as ISA [40], Samba [43], CC [44], OPSM [45], xMotif [46], and Hierarchical Clustering.

### Assessing robustness of biDCG in presence of overlaps: Synthetic data 2

The second artificial scenario is designed to study the behavior of biDCG with respect to increased interaction complexity. It is a repeat of the scenario 1 described above, with the main difference that a single gene may be activated by 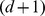 transcription factors and in each condition 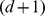 transcription factors can be active, where 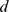 is defined as the overlap level (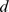 was set to 0 in scenario 1). This increase in regulation complexity leads to overlaps of the implanted transcription modules, i.e. of the biclusters. The corresponding datasets are expression matrices 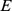 of size 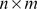 with 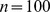 and 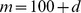 that contain 10 possibly overlapping biclusters. We still consider the two types of expression matrices used in scenario 1, namely those with constant biclusters and those with additive biclusters. All experiments are performed in the absence of additional white noise; note that by construction the matrices mimicking additive biclusters do contain noise.

**Figure 3:**
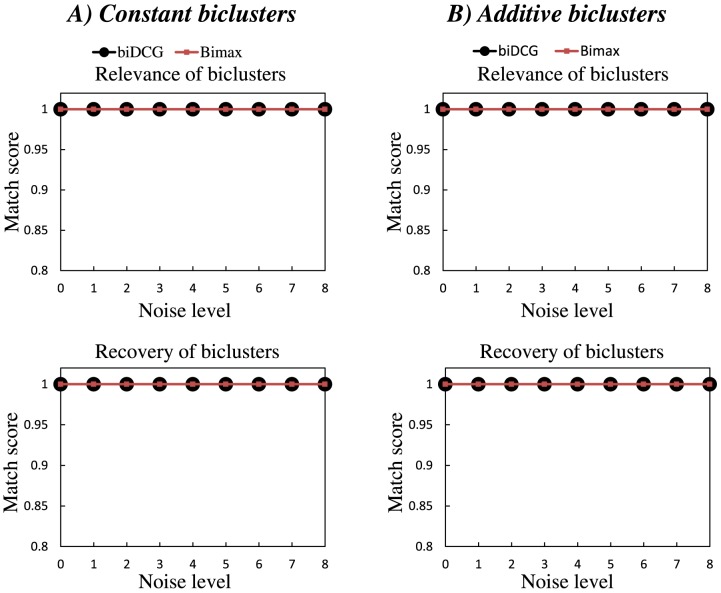
Effects of overlaps on the relevance and recovery levels of biclusters identified by biDCG and Bimax. The biclustering techniques biDCG and Bimax [19] were applied on synthetic expression matrices designed to represent 10 biclusters, either constant (left panels, A and C), or additive (right panels, B and D). In both cases, the average relevance (i.e. the extent with which a generated bicluster represent a true bicluster) and the average recovery level (i.e. the extent with which true biclusters are recovered) are plotted as a function of the overlap level introduced in the expression matrices.

Figure 3 depicts the recovery level and relevance of the biclusters found by biDCG and Bimax on the data matrices generated for scenario 2 for different overlap levels. biDCG performs well at all overlap levels as it recovers all hidden modules. As such, it performs as well as Bimax. Since the data used are were generated the same way than the data generated by Prelić and co-workers, we can also say that it compares favorably to the other biclustering techniques they have tested, as observed when comparing Figure 3 in this paper with Figure 2 of reference [19]). In particular biDCG performs significantly better than traditional hierarchical clustering methods, highlighting the advantage of an explicit biclustering technique.

**Figure 4:**
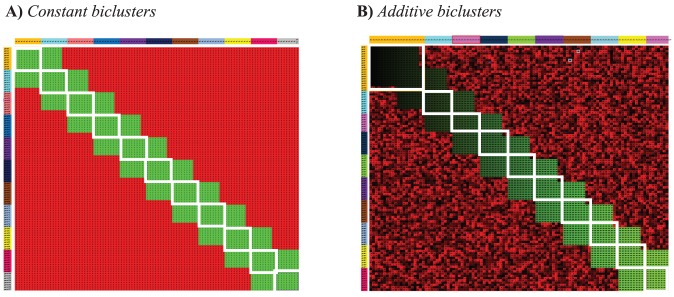
Iterative refinements of the biclusters identified by biDCG. The biclustering method biDCG was applied on two synthetic expression matrices designed to represent 10 biclusters, either constant (left panel, A), or additive (right panel, B), both with overlap of 8 between the biclusters (see text for details). The initial biclusters (shown as white boxes) defined by simple applications of DCG on the whole matrix do not match correctly with the biclusters that were implanted; for example, DCG identified 11 biclusters in the constant cluster case (panel A). Iterative refinements of the biclusters however lead to the correct identification of all 10 reference biclusters, as shown as green sub matrices.

One of the key features of biDCG is to iteratively refine the definitions of the clusters along the subject and feature dimensions, based on the dual relationships found between their subgroups (the biclusters). This iterative procedure greatly improves the performance of biDCG, as illustrated in Figure 4. We considered two expression matrices used in the analysis described above, one for constant and one for additive clusters, both with a large overlap level (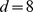). Naive analyses of these two matrices using DCG (i.e. without iterative refinements of the biclusters) lead to inexact identification of the transcription modules. For example, DCG identified 11 biclusters for the constant clusters (shown as white boxes in Figure 4A), while the matrix was generated with only 10 transcription modules. Iterative refinements of the biclusters however lead to modifications of the cluster definition and repositioning of the biclusters whose converged positions match with the actual transcription modules for both the constant and additive cluster cases (Figure 4).

We note that the test cases presented above relate to biclusters overlapping over the diagonal of the heat map matrix. In situation where overlaps would occur off-diagonal, we expect biDCG to still recover the actual biclusters. The biDCG procedure iterates alternatively in the row and column directions; as long as the non-overlapping parts are not too small, biDCG is expected to identify the differences among all involving row and column vectors of any overlapping region, leading to their resolution.

### Analyzing different types of lung tumors: Real dataset A

Synthetic data sets are inherently biased as they rely on an artificial model with usually well behaved noise. As such, they cannot fully reflect the actual behavior of an algorithm on a real biological dataset. Therefore, we tested biDCG on a real, published dataset of gene expression patterns for cancer affected and healthy patients [39]. This dataset comes from a study that includes data on 203 patients, out of which 186 were affected by five types of lung cancer, namely adenocarcinoma (AD, 127 patients), squamous cell lung carcinomas (SQ, 21 patients), pulmonary carcinoids (COID, 20 patients), small cell lung carcinomas (SCLC, 6 patients), and other adenocarcinomas (12 patients that were suspected to suffer from extra pulmonary metastases based on clinical history), and 17 healthy patients with normal lungs (NL). The original study included expression data for 3,312 genes [39]; out of those 1543 were selected as being the most informative [23]. We note that in this data set, the AD patients represent a very large majority, likely containing many subtypes. This heterogeneity may have adverse effects on the clustering procedures as it could blur the geometric structure of the data. To alleviate this problem, we divided this dataset into two subgroups, following the partition already considered in the original study [39]. Namely, we considered a dataset A containing all patients except those affected by AD, each characterized with the the expressions of all 1543 genes mentioned above, and a dataset B that contains 65 AD patients characterized with the expressions of a reduced set of 675 genes. Results for the latter are presented in the following subsection; here we focus on dataset A.

**Figure 5:**
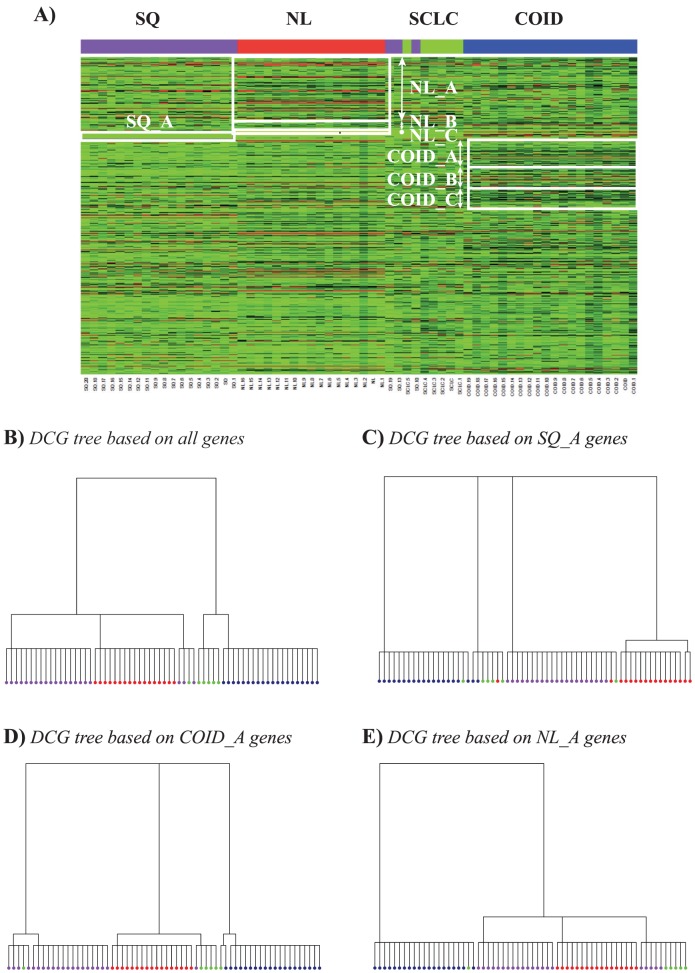
BiDCG analysis of lung cancer data. The set of patients described in Bhattacharjee et al. [39] include 21 patients with squamous cell lung carcinomas (SQ), 20 patients with pulmonary carcinoids (COID), 6 patients with small cell lung carcinomas (SCLC), and 17 healthy patients with normal lungs (NL). Gene expression patterns over 1543 relevant genes were collected for each patient. The biDCG procedure applied to these data identified 7 biclusters, marked in white on the specially constructed heat map shown in panel **A**. Bicluster SQ_A for example identifies a set of genes, named also SQ_A, that best identifies patients with SQ lung cancers. Similarly, the three subsets of genes NL_A, NL_B, and NL_C can be thought as containing signature genes for healthy patients, while the subsets of genes COID_A, COID_B, and COID_C contain genes that identify best COID patients. Panel **B** shows the DCG tree on all patients based on all genes, while panels **C**, **D**, and **E** show the equivalent DCG trees based on the gene subsets SQ_A, COID_A, and NL_A, respectively. The color coding for the DCG trees is: purple, SQ, red, NL, green SCLC, and blue, COID.

Using the Spearman's correlation coefficient between the expression vectors covering all 1543 genes mentioned above as a distance measure between two patients, we computed first a DCG analysis for all patients in dataset A. The corresponding tree is shown in Figure 5B; a similar tree was already presented in our previous work [36]. At the lower level, it contains 5 clusters, four of which are pure, i.e. each of these four clusters only includes patients with one specific type of lung cancer. We will refer to these four pure clusters as SQ, COID, SCLC, and NL, even though they do not match exactly the actual patient types, i.e. there is a 0.94 relevance score for the clusters identified, and a 0.94 recovery score for the actual patient subgroups (see Methods for a definition of these scores).

The only mixed cluster includes three patients with SQ and one patient with SCLC. At a higher level in the tree, the patients are divided into two conglomerate clusters, one with COID and SCLC subtypes, the other including patients with SQ and NL. The merging of the two subtypes COID and SCLC into one conglomerate cluster indicates that patients with these two types of cancers are “closer” to each other than to patients with either SQ or NL cancer types. We should note however that the concept of close relates here to the use of the Spearman's correlation coefficient; another distance measure may have led to other conglomerate clustering.

We performed a full biDCG analysis in which we included all non-AD patients from the original 203 patients, and all 1543 relevant genes. The final heat map is shown in Figure 5A. Our primary focus is on the three categories NL, COID and SQ, as the smallest category, SCLC contains only 6 patients. biDCG identified a total of 7 biclusters (or dual relationships) for these three categories, one for the SQ patients, three for the healthy patients (NL), and three for the COID patients. Each of these biclusters defines a set of genes that is most pertinent to one type of patients. To illustrate the relevance of the information produced by biDCG, we generated DCG trees over all patients, including only subsets of genes identified within the biclusters. DCG trees based on subsets relevant to SQ (SQ_A), COID (COID_A), and NL (NL_A) are shown in Figure 5C, D, and E, respectively.

The DCG tree based on the SQ_A gene subset is expected to provide a good separation of the SQ patients. Indeed, as observed in Figure 5C, these patients are now part of the same cluster, while they were divided into two clusters in the DCG tree based on all genes (see discussion above). The three other types of patients remain reasonably well partitioned within this SQ-specific tree, although there are more outliers than in the DCG tree based on all genes. In the DCG tree based on the COID_A gene subset (Figure 5D), all COID patients remain regrouped. Interestingly, one SCLC patient is now regrouped with these patients. Again the three other types of patients remain well regrouped. The DCG tree based on the NL_A gene subset maintains all healthy patients together in the same cluster. Interestingly, there is more mixing of the other patients within this tree, indicating that genes that can act as signature of patients free of cancer would not be good signatures of lung cancer type.

We note also that the iterative refinement of the biclusters performed within biDCG lead to an improved definition of the patient subgroups, as the relevance score for the clusters identified increase from 0.94 to 0.96, with the same improvement for the recovery score for the actual patient subgroups.

The dual relationships or biclusters defined above identify a set of genes that can be considered as markers for a certain phenotype, should it be healthy or with a specific type of lung cancer in the case considered here. The question arises as to the biological relevance of these putative markers. Ideally, the gene markers identified for a phenotype should be connected to this phenotype through experimental evidence. In practice however, such data on the direct relationship between a gene and a disease is often not available. As an ersatz to such knowledge, it is possible to test a group of genes for possible enrichment in a given characteristic, which would indicate that these genes form an homogeneous group and share (at least one) similar function. We correspondingly tested the set of genes identified as markers for SCLC, SQ, COID and NL against the Gene Ontology (GO) [47] using two software packages designed to assess such gene clusters, namely the Gene Ontology AnaLyzer (GOAL) [48] and gene annotation tool associated with the Database for Annotation, Visualization and Integrated Discovery (DAVID) [49]. Note that DAVID includes more reference terms than GOAL, as it extends beyond the Gene Ontology [49]. In this analysis, a group of gene is considered “enriched” if at least one reference term from GO (or another library considered by DAVID) is enriched with a P-value better than 0.05 (where the Benjamini and Hochberg multiple test correction has been applied [50]). Results are given in Table 1. We found that all gene subgroups associated to SCLC, SQ, COID, and NL patients, respectively, are considered enriched by DAVID, with only the three subgroups COID_A, COID_C, and NL confirmed enriched by GOAL. The differences between GOAL and DAVID may only be a reflection of the different set of reference terms they include. It remains that these results hint that the biDCG method identified biclusters where the genes corresponding to one patient subgroup have at least one statistically significant common biological characteristics.

**Table 1:** Dataset A: biclusters significantly enriched by any GO Biological Process category.

Bicluster a	# of genes	# of enriched terms b	# of enriched terms c
		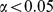 (GOAL)	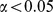 (DAVID)
SQ_A	38	0	10
COID_A	134	1	1
COID_B	101	0	8
COID_C	98	1	1
NL d	365	80	29
SCLC	35	0	5

a Biclusters identified by biDCG, as marked on Figure 5.

b Number of GO terms enriched in the gene set, with a significance level better than 0.05: GOAL [48] results.

c Number of functional terms enriched in the gene set, with a significance level better than 0.05: DAVID [49] results.

d We regrouped all genes from NL_A, NL_B, and NL_C, as those correspond to healthy patients.

### Analyzing different subtypes of a specific lung tumor type: Real dataset B

The gene expression data from Bhattacharjee et al. [39] also contained information about 65 patients suffering from lung adenocarcinoma (AD). In their paper Bhattacharjee et al. [39] performed a clustering analysis of these patients plus the 17 healthy patients using the probabilistic model-based clustering method implemented in AUTOCLASS [51]. They identified five subgroups of AD patients that they could relate to phenotypes: four subclasses of primary lung adenocarcinomas (clusters C1 to C4), and one subclass corresponding with patients with normal lung but putative colon cancer metastases (cluster CM). The DCG tree based on the AD patients also identifies five main clusters that match with the clusters defined above, plus one cluster comprised of mixed patients that originally included patients from the four subclasses of primary lung adenocarcinomas identified by Bhattacharjee et al. [39]. We refer to these five clusters as C1′, C2′, C3′, C4′, and CM′, based on their degree of overlaps with the original subgroups. We note however that in the DCG analysis, there are some overlaps between subclasses C1′ and CM′. These overlaps, as well as the smaller clusters observed by DCG may correspond to the smaller, less stable groups identified in the original study by Bhattacharjee et al. [39]. These differences translate into relatively poor relevance and recovery scores of 0.62 and 0.69, respectively.

**Figure 6:**
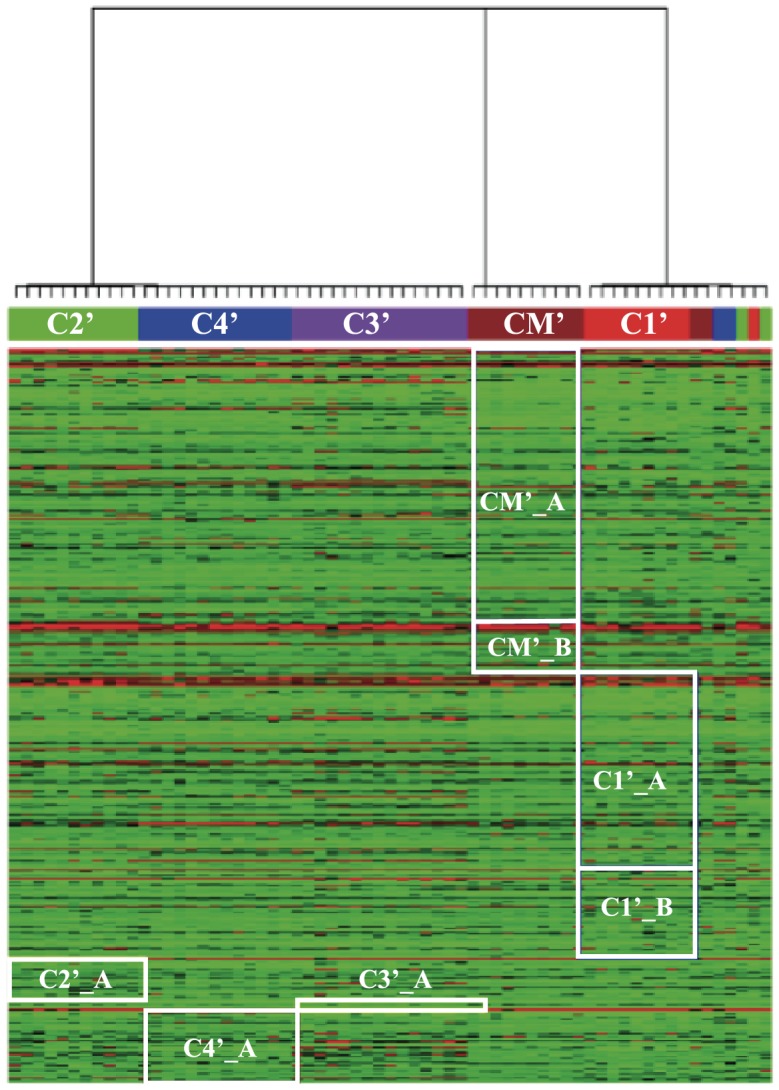
BiDCG analysis of lung cancer data for patients with adenocarcinoma (AD). We consider 65 patients with AD from the dataset described in Bhattacharjee et al. [39]. Gene expressions of 675 relevant genes are available for each patient. The biDCG procedure applied to these data identified 7 biclusters, marked in white on the specially constructed heat map. Each of these biclusters identifies a set of genes that can serve as signature for a specific type of patients, a so-called dual relationship.

We performed a full biDCG analysis in which we included all 65 AD patients and all 675 relevant genes. The final heat map is shown in Figure 6. biDCG identified a total of 7 biclusters, two for each of the two patient subgroups C1′ and CM′, and one for each of the patient subgroups C2′, C3′, and C4′. In parallel, biDCG leads to a modification of the memberships within the five clusters C1′, C2′, C3′, C4′, and CM′, leading to relevance and recovery scores of 0.81 compared to the subclasses defined in [39]. The improved match between the two sets of subclasses is a good indication that biDCG is capturing relevant information from the data, as these subclasses were carefully validated in the original study.

We assessed the biological relevance of the biclusters identified by biDCG using GOAL [48] and DAVID [49], using the same protocol described above for dataset A. Results are given in Table 2. We found that all biclusters are significantly enriched by at least one biological category according to either GOAL or DAVID, and for most biclusters by both (the only three exceptions are C1′_B and CM′_B that are only found enriched by DAVID, and reversely C3′_A that is found to be enriched by GOAL only). Just like for dataset A, these results place emphasis on the fact that biDCG is able to retrieve biologically relevant information. Finally we note that biDCG finds the highest proportion of enriched biclusters on these two real data sets when compared to other biclustering techniques such as Bimax [19], Plaid [52], CC [44], and xMotif [46], as illustrated in Table 3.

**Table 2:** Dataset B: biclusters significantly enriched by any GO Biological Process category.

Bicluster a	# of genes	# of enriched terms^b^	# of enriched terms^c^
		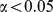 (GOAL)	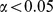 (DAVID)
C1′_A	372	51	114
C1′_B	170	0	32
C2′_A	78	3	2
C3′_A	18	1	0
C4′_A	141	10	11
CM′_A	518	109	172
CM′_B	99	0	14

a Biclusters identified by biDCG, as marked on Figure 6.

b Number of GO terms enriched in the gene set, with a significance level better than 0.05: GOAL [48] results.

c Number of functional terms enriched in the gene set, with a significance level better than 0.05: DAVID [49] results.

**Table 3:** Proportion of biclusters significantly enriched by any GO Biological Process category for four biclustering methods. Results are shown for datasets A and B (see text for details).

Dataset A			
Algorithm	# of biclusters identified	# of enriched biclusters a	Proportion of enriched biclusters (%)
biDCG	6	6	100
Bimax [19]	8	5	62.5
Plaid [52]	8	5	62.5
xMOTIFs [46]	8	5	62.5
CC [44]	8	2	25

Dataset B			
Algorithm	# of biclusters identified	# of enriched biclusters a	Proportion of enriched biclusters (%)
biDCG	7	6	85.7
Bimax	10	5	50
Plaid	4	3	75
xMOTIFs	4	2	50
CC	5	1	20

a Biclusters are considered enriched if any GO term was enriched with a P-value better than 0.05 after multiple test correction.

### Concluding remarks

Biclustering techniques have become very popular in cancer genetics studies, as they are tools that are expected to connect phenotypes to genotypes, i.e. to identify subgroups of cancer patients based on the fact that they share similar gene expression patterns as well as to identify subgroups of genes that are specific to these subtypes of cancer and therefore could serve as biomarkers. The relationships between such patient subgroups and gene subgroups are referred to as bicluters. Biclustering techniques are not yet fully mature and there are still many new such techniques that are developed. The recent literature on this topic makes no secrets of their limitations and problems. Some of these problems relate to the treatment of noise, to the absence of a unifying definition of the merit function that evaluates the quality of biclusters, as well as to the choice of the clustering techniques used to reveal these biclusters.

In this paper we proposed a new approach for identifying biclusters in gene expression matrices that is designed to alleviate at least some of these problems. This method, biDCG, rests on two key concepts. First, it is important to capture the geometry of the data (where data relates indifferently to the cancer patients or the genes whose expressions are assessed), i.e. to identify robustly their substructures. We use our own new clustering method, DCG-tree that generates an ultrametric topological space on the data [36]. It has two main features that are keys to the success of biDCG. First, the ultrametric space is less sensitive to noise in the data, and second, it has a built-in mechanism for self-correcting clustering membership across different tree levels [36]. The second key concept is that biDCG optimizes the definitions of bicluster membership through an iterative reclustering procedure that is designed to identify consistent and robust relationships between patients and gene expression. We have validated biDCG both on simulated and real data. Based on the simulated data we have shown that biDCG compares favorably to other biclustering techniques applied to cancer genomics data. The results on the real data sets have shown that biDCG is able to retrieve relevant biological information.

There is still much room for improvement within biDCG. For example, while DCG has a built-in mechanism to convert the metric used to compare gene expression data into a ultrametric, the quality of this conversion cannot be dissociated from the quality of the original metric. We have used the Spearman's correlation coefficient for this purpose. Its usage for comparing gene expression profiles of two patients is quite intuitive. It captures similar shapes of the expression profiles, ignoring differences in magnitude. Its usage however to compare the expression of two genes over a range of patients is less intuitive. This issue was already discussed [28]; we believe it still needs to be revisited. In addition, the final heat map currently generated by biDCG provides a visual representation of the biclusters that are identified. It does not provide any visual information however on the strength and relevance of the bicluster memberships. We also note that it is not fully clear which clusters identified by biDCG are meaningful. We have used GOAL and DAVID to assess the relevance of the biclusters, based on the idea that an enrichment in a GO term within a gene group is likely to indicate that these genes relate to a similar biological function. While our results are insofar interesting in that respect, the problem of selecting the most relevant biclusters still need to be considered for further analysis. We plan to work on these issues in future studies.
